# Exploiting nanotechnology to target cancer

**DOI:** 10.1038/sj.bjc.6603707

**Published:** 2007-04-03

**Authors:** S Sengupta, R Sasisekharan

**Affiliations:** 1 BWH-HST Center for Biomedical Engineering, Department of Medicine, Brigham and Women's Hospital, Cambridge, MA 02139, USA; 2 Biological Engineering Division, Massachusetts Institute of Technology, Cambridge, MA 02139, USA; 3 Harvard-MIT Division of Health Sciences and Technology, Cambridge, MA 02139, USA

## Abstract

Nanotechnology is increasingly finding use in the management of cancer. Nanoscale devices have impacted cancer biology at three levels: early detection using, for example, nanocantilevers or nanoparticles; tumour imaging using radiocontrast nanoparticles or quantum dots; and drug delivery using nanovectors and hybrid nanoparticles. This review addresses some of the major milestones in the integration of nanotechnology and cancer biology, and the future of nanoscale approaches for cancer management.

‘The principles of physics, as far as I can see, do not speak against the possibility of manoeuvreing things atom by atom’ – Feynman (1959). Since Feynman's talk at the annual meeting of the American Physical Society in December 1959, we have made significant progress in enabling nanotechnology for revolutionising medicine. In cancer biology this has translated into major advances in primarily three areas: early detection, tumour imaging and drug delivery. In this article, we highlight some of the key nanotechnologies in each of these three areas, with a brief discussion of the challenges and advances for each approach.

## DRIVERS FOR DEVELOPMENTS IN NANOTECHNOLOGY

The drivers for advances in nanotechnology have been the development of tools that enable us to manipulate at an atomic scale, and materials and chemistry that allow the constructions of novel structures. Although it is not the focus of this review to address these tools in details, it is important to realise potentials of these analytical and fabrication tools, which if integrated with fundamental biology can provide essential breakthroughs in the fight against cancer.

It was originally the electron microscope, with scanning or transmission capabilities, which was used to image at the nanoscale range, but it was limited by the steps involved in the preparation of the object to be imaged. More recently, scanning probe devices have been enormously successful in biological manipulations at the single molecule level (Engel *et al*, 1999). An interesting adaptation of the scanning probe devices has been the Scanning Tunneling Microscope, which uses ‘tunnelling’ or the flow of electrons from a probe tip to a charged surface and *vice versa* to generate the contours of a surface (Horber and Miles, 2003), or to move a structure atom by atom. Another modification is the use of the cantilever technology to detect tumour markers (Wu *et al*, 2001). Furthermore, the scanning probe device has also been integrated with wet lithography techniques in an approach called the dip pen nanolithography, to print designer nanoscale structures in three dimensions (Piner *et al*, 1999).

## NANOTECHNOLOGY AND BIOSENSORS

The successful outcome of cancer chemotherapy often depends on the early detection of the cancer lesion. However, in most cases the transformation remains undetected until clinical manifestations, arising primarily from the absence of highly sensitive techniques that can detect low levels of markers. Furthermore, clinically relevant markers, such as prostate serum antigen (PSA), have a broad range on baseline expression within the population, which makes them notoriously bad predictors of future events. Furthermore, in certain cases, a profile of multiple markers may shed more light on the cancer status than a single marker.

The limitation of sensitivity can be resolved by reducing the dimensions to the nanoscale. Using the dip-pen nanolithography approach, Mirkin's group printed a nanoscale oligonucleotide chip on both metallic and insulating substrates, which exhibited the sequence-specific binding properties of the DNA (Demers *et al*, 2002). This ‘bottom-up’ approach can enable the detection of markers at very low concentrations, especially when integrated with another emerging technology, the nanocantilevers.

**Figure 1:**
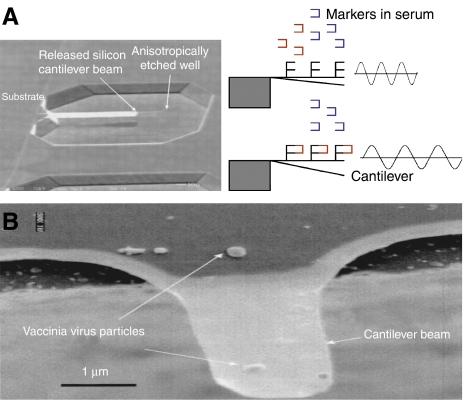
(**A**) s.e.m. of a cantilever. Reused with permission from A Gupta, D Akin and R Bashir, J of Vaccum Science and Technology B, 22, 2785 (2004). Copyright 2004, AVS The Science and Technology Society. (**B**) Schematic showing the functionalisation of the cantilever such that it can bind to a cancer marker. The cantilever can be set to a defined frequency that changes following the binding of a marker. Reducing the cantilever to the nanoscale size can enable highly sensitive detection of cancer markers. Reused with permission from A Gupta, D Akin and R Bashir. Applied physics letters, 84, 1996 (2004), copyright 2004, American Institute of Physics.

The nanocantilevers-based sensors operate on the concept that biomolecular binding events will deflect the nanocantilevers resulting in a change in their resonating frequencies (Fritz *et al*, 2000) (Figure 1). The nanoscale dimensions offer the possibility of designing structures with multiple such components, each with distinct detection capabilities, which can allow the simultaneous profiling of a range of predictors for a given cancer. Furthermore, the reduction in the dimension also holds the promise of increasing the sensitivity to a scale where it is possible to detect subtle changes in the profile. For example, using a microscale cantilever, Hansen *et al* (2001) reported that it was possible to discriminate DNA single-nucleotide mismatches. In a separate study, Wu *et al* (2001) demonstrated the feasibility of the approach to quantify PSA at a clinically relevant concentration. Although both these studies were done using a microscale cantilever, it is not difficult to envisage a future where nanowires and nanotubes will function as the sensors (Zheng *et al*, 2005), offering the possibility of large-scale monitoring of the proteome or the transcriptome for subtle changes in the profile and relate it to the early stages of cancer progression.

Another emerging area is the use of nanoparticles for diagnostics (Cuenca *et al*, 2006). For example, Mirkin and co-workers developed a system that relies on nanoparticle probes encoded with DNA that are unique to the protein target of interest, and antibodies that can sandwich the target captured by the probes. Magnetic separation of the target–probe complex followed by dehybridisation of the oligonucleotides on the nanoparticle probe surface allows the determination of the presence of the target protein. Using this approach, they could attain substantial amplification as each nanoparticle probe carried with it a large number of oligonucleotides per protein binding event. Adding a polymerase chain reaction on the oligonucleotide bar codes could boost the sensitivity of detecting PSA to 3 aM which is six orders of magnitude more sensitive than the clinically accepted conventional assays (Rosi and Mirkin, 2005). In other studies, gold nanoshell-based immunoassays that change colour following interactions with the ligand and quantum dots labelled with targeting antibodies have been used to detect specific tumour markers. These offer significant advantages of stability and ‘tunability’ over traditional staining techniques; in particular, they do not photobleach and can be designed to emit different colours based on their sizes.

## NANOTECHNOLOGY AND TUMOUR IMAGING

Although the use of nanotechnology has generated excitement in the area of tumour biology, especially enabling the simultaneous monitoring of multiple cellular markers based on the tunability of the nanostructures, another application that is achieving major milestones towards clinical applicability is the use of nanoparticles to image tumours *in vivo*.

**Figure 2:**
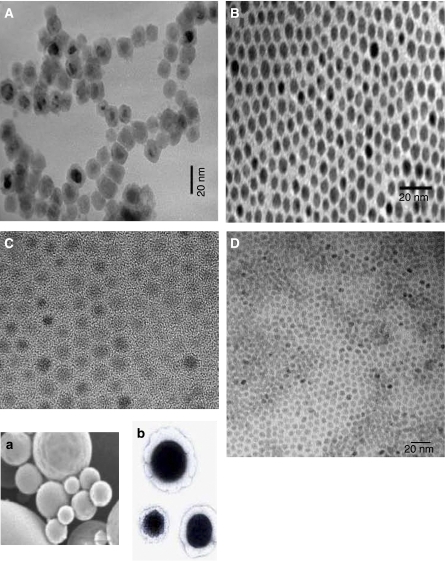
Electron micrographs of different types of nanoparticles. (**A**) Fe nanoparticles coated with poly(*N*-vinyl-2-pyrrolidone), which stabilises the nanoparticles. (**B**) TEM image of Au nanoparticles, (∼6 nm), which are being harnessed for sensing protein markers. The aggregation of these nanoparticles is visualised from a change in absorbance; (**C**) TEM image for Fe_3_O_4_ nanoparticles (size ∼5 nm), which are being developed as radiocontrast agents. (**D**) TEM image for CdSe quantum dots (size ∼4 nm). The size-dependent optical tunability of quantum dots make them ideal candidates for imaging. Courtesy of Dr Zhi-Hui Ban, MIT. **Inset** shows electron micrographs of a nanocore and a nanocell. (**a**) Scanning electron micrograph of a nanocore synthesised from doxorubicin-conjugated PLGA polymer. (**b**) Transmission electron micrograph of a cross-section of a nanocell. The dark centre is the nanocore entrapped inside the lipid layer. The hybrid two-chambered nanoparticle displays a spatiotemporal release kinetics, rapidly releasing an antiangiogenesis agent from the outer layer follwed by a delayed release of a chemotherapeutic agent. The release of the antiangiogenesis agent causes a vascular collapse entrapping the chemotherapy-loaded nanocore within the tumour. As the tumour becomes hypoxic, the nanocore degrades, focally releasing the chemotherapy agent. (Sengupta *et al*, 2005).

Iron oxide nanocrystals (Figure 2) with superparamagnetic properties have been used as contrast agents in magnetic resonance imaging (MRI) as they cause changes in the spin–spin relaxation times of the neighbouring water molecules (Moghimi *et al*, 2005). In an interesting study, highly lymphotropic superparamagnetic nanoparticles, which gain access to lymph nodes by means of interstitial–lymphatic fluid transport, were used in conjunction with high-resolution MRI to reveal small and otherwise undetectable lymph-node metastases in patients with prostate cancer (Harisinghani *et al*, 2003).

Quantum dots, which are generally defined as particles with physical dimensions smaller than the exciton Bohr radius (Chan *et al*, 2002), are emerging as powerful optical contrast agents, both for monitoring cellular events and for imaging tumours *in vivo*. Quantum dots (Qdots) offer unique size- and composition-tunable fluorescence emission from visible to infra-red wavelengths, large absorption coefficients across a wide spectral range and greater phostability and signal intensity. Initial concerns regarding nonselective uptake of Qdots into the reticuloendothelial system and solubility were overcome using pegylation chemistry (Akerman *et al*, 2002), which results in a hydrophilic surface. Furthermore, coating with a targeting peptide resulted in the selective delivery to the target organs such as tumour vasculature or the lungs (Akerman *et al*, 2002).

Quantum dots have now been used track signalling pathways, such as the erbB/HER receptor-mediated signalling (Lidke *et al*, 2004), cell–cell interaction, such as in breast tumour (Alivisatos *et al*, 2005), and for spectrally distinguishing multiple species within the tumour *milieu in vivo* (Stroh *et al*, 2005). Furthermore, labelling the tumour cells with Qdots also enabled the tracking of metastatic tumour cell extravasation (Voura *et al*, 2004). The last study also established the safety profile of Qdots *in vivo*, although extensive safety and pharmacokinetics studies need to be carried out before use in humans given the fact that Qdots are composed of heavy metals (such as Cd), which can induce liver and kidney damage. However, one should also consider that before biological use the metals are sequestered inside an organic shell, and are administered at doses much below the known toxic levels of the metals.

The advantages of using Qdots over traditional radiocontrast dyes lies in the avoidance of harmful radiations that are traditionally used. The Qdots that emit at longer wavelengths are especially suitable for *in vivo* imaging as there is low tissue scattering and absorption in this range. However, the goal is to preserve the optical properties of these Qdots following surface modifications to make them biocompatible (Jiang *et al*, 2004). Emergence of newer technologies that enable biocompatible Qdots with narrow emission spectral range and enhanced photostability – and their integration with improved pattern recognition and image analysis techniques – will revolutionise the tumour imaging field.

## NANOTECHNOLOGY AND TUMOUR DRUG DELIVERY

One of the earliest applications of nanotechnology in medicine was the use of liposomes in the nanoscale range to deliver chemotherapy payloads to the tumour (Moghimi *et al*, 2005). Liposomal formulations of doxorubicin are now approved for use in Kaposi sarcoma, breast cancer and refractory ovarian cancer. An advantage that the liposomes provided is the delivery in an aqueous phase, which avoided the use of solubilising agents such as Cremophor that are associated with hypersensitivity reactions.

A major milestone in drug delivery systems engineering was the development of technologies that can mask the nanodelivery carriers from the immune system. Significant modifications have been introduced in the composition of the lipid bilayers that have optimised the ‘stealth’ capability and also affected the tissue-specific homing. For example, the introduction of synthetic lipid derivatives of polyethylene, where the hydrophilicity of the PEG chains confers ‘stealth’ capability to the liposomes (Allen *et al*, 1991). These liposomes remain in the circulation for prolonged periods of time and accumulate passively in the tumours via an enhanced permeation and retention (EPR) effect (Yuan *et al*, 1994). The EPR effect arises from the unique morphology of the tumour vasculature, which is highly leaky with pore sizes as large as 600 nm that allows the nanoscale liposomes to easily extravasate out into the tumour. Furthermore, the limited lymphatic drainage prevents the clearance from the tumours. Passive targeting through the EPR effect results in delivery of 3–10 times more drug to solid tumours as compared to free drug. Indeed, the clinical liposomal formulations of doxorubicin were shown to deliver between 5 and 11 times more doxorubicin to Kaposi's sarcoma lesions than to normal skin, leading to an overall tumour response rate as high as 80%. However, selective delivery can be further improved upon by integrating targeting technologies to the delivery platform. One of the attractive targets for ‘homing’ has been the dividing endothelium, which is a hallmark of the tumour vessels. For example, a nanoparticle carrying a plasmid DNA encoding a dominant-negative mutant form of RAF kinase was reported to exert antiangiogenesis effects when targeted against a_v_*β*_3_ integrins (Hood *et al*, 2002). There are still bottlenecks in the existing ‘homing’ technologies, such as in the case of targeting TNP470-immunoliposomes using a single-chain variable fragment against human endoglin on angiogenic endothelial cells resulted in neurotoxicity at higher doses, which indicated failure of the targeting mechanism. This emphasises the need for novel targeting strategies, such as using aptamer (Farokhzad *et al*, 2006).

Another emerging approach for specifically targeting the tumour cells without collateral damage is the polymer-directed enzyme prodrug therapy, which uses a combination of a polymeric prodrug and polymer-enzyme conjugate to generate a cytotoxic drug rapidly and selectively within the tumours. Several polymer-cyotoxic drug conjugates are already in early clinical trials, including N-(2-hydroxypropyl) methacrylamide (HPMA) copolymer-doxorubicin, paclitaxel, HPMA-platinate and PEG-campothecin. Recently, a conjugate of TNP470 and HPMA was also shown to be an effective antiangiogenesis therapy (Satchi-Fainaro *et al*, 2004), homing into tumours through the EPR effect with a concomitant reduction in toxicity. The advantages of the HPMA copolymer were also the presence of multivalent side chains that permits a higher loading of drugs on the polymer and the reduced immunogenic profile that allows repeated dosing.

Approaches based on the conjugation of chemotherapeutic agents with nanoparticle-forming biodegradable polymers have long been developed (Sengupta *et al*, 2005), avoiding the ‘burst release’ associated with nanoparticles. However, the limitation of using polymers such as PLGA remains in the limited number of valent sites to which a chemotherapeutic agent could be coupled. An emerging approach to overcome this limitation is the use of dendrimers, which are synthetic, highly branched, symmetrical macromolecules. Altering the terminal functionalities of the dendrimers (such as PAMAMs and PPIs) enables targeted drug delivery, whereas the interior cavities can be used for loading both hydrophobic and hydrophilic drugs. However, further characterisation of the dendrimers is needed to determine their safety profile before they can reach the clinics.

## FUTURE DIRECTIONS

Although most of the technologies have focused on the delivery of single chemotherapeutic agents to the tumours, it is increasingly becoming clear that an integrative approach may work better than a reductionist approach. Nanotechnology platforms can provide the unique niche within this space by enabling multimodal delivery with a single application. For example, in a recent study, we demonstrated that the spatiotemporal release of an antiangiogenesis agent and a chemotherapeutic agent from a hybrid nanoparticle, a ‘nanocell’ (Figure 2) could exert a better therapeutic outcome as compared with existing delivery approaches (Sengupta *et al*, 2005). A nanocell comprises of a pegylated phospholipid outer layer entrapping a nanocore. An antiangiogenic agent is partitioned into the lipid layer, whereas a chemotherapeutic agent is conjugated to a biodegradable polymer to generate the nanocores. The nanocell homes into the tumour vasculature through the EPR effect and releases the antiangiogenic agent, resulting in the collapse of the tumour vasculature. The chemotherapy agent is then slowly released from the entrapped nanocores, inducing apoptosis and overcoming the potential for hypoxia-induced ‘reactive resistance’.

Another emerging direction in the application of nanotechnology in cancer is the use of self-assembly techniques, such as to design monodisperse delivery vehicles like polymerosomes (Xu *et al*, 2005) or to develop sensors. Nanotechnology is also enabling highly efficent radiotherapy, such as the injection of single doses of an atomic nanogenerator at kilobecquerel (nanocurie) levels into mice bearing solid prostate carcinoma or disseminated human lymphoma induced tumour regression and prolonged survival, without toxicity, in a substantial fraction of animals (McDevitt *et al*, 2001). In another study, metal nanoshells with tunable optical resonance were shown to induce irreversible thermal damage to tumour cells when exposed to near infrared light (Hirsch *et al*, 2003).

Nanotechnology clearly holds immense potential for targeting cancer. These approaches will encompass the desired goals of early detection, tumour regression with limited collateral damages, and efficient monitoring of response to chemotherapy. The exciting milestones made in these areas need to be paralleled with safety evaluations of the platforms before they are translated to the clinics. Nevertheless, we believe that the next few years are likely to see an increasing number of nanotechnology-based therapeutics and diagnostics reaching the clinic.

## References

[bib1] Akerman ME, Chan WC, Laakkonen P, Bhatia SN, Ruoslahti E (2002) Nanocrystal targeting *in vivo*. Proc Natl Acad Sci USA 99: 12617–12621 12235356 10.1073/pnas.152463399 PMC130509

[bib2] Alivisatos AP, Gu W, Larabell C (2005) Quantum dots as cellular probes. Annu Rev Biomed Eng 7: 55–76 16004566 10.1146/annurev.bioeng.7.060804.100432

[bib3] Allen TM, Hansen C, Martin F, Redemann C, Yau-Young A (1991) Liposomes containing synthetic lipid derivatives of poly (ethylene glycol) show prolonged circulation half-lives *in vivo*. Biochim Biophys Acta 1066: 29–36 2065067 10.1016/0005-2736(91)90246-5

[bib4] Chan WC, Maxwell DJ, Gao X, Bailey RE, Han M, Nie S (2002) Luminescent quantum dots for multiplexed biological detection and imaging. Curr Opin Biotechnol 13: 40–46 11849956 10.1016/s0958-1669(02)00282-3

[bib5] Cuenca AG, Jiang H, Hochwald SN, Delano M, Cance WG, Grobmyer SR (2006) Emerging implications of nanotechnology on cancer diagnostics and therapeutics. Cancer 107: 459–466 16795065 10.1002/cncr.22035

[bib6] Demers LM, Ginger DS, Park SJ, Li Z, Chung SW, Mirkin CA (2002) Direct patterning of modified oligonucleotides on metals and insulators by dip-pen nanolithography. Science 296: 1836–1838 12052950 10.1126/science.1071480

[bib7] Engel A, Gaub HE, Muller DJ (1999) Atomic force microscopy: a forceful way with single molecules. Curr Biol 9: R133–R136 10074420 10.1016/s0960-9822(99)80081-5

[bib8] Farokhzad OC, Cheng J, Teply BA, Sherifi I, Jon S, Kantoff PW, Richie JP, Langer R (2006) Targeted nanoparticle-aptamer bioconjugates for cancer chemotherapy *in vivo*. Proc Natl Acad Sci USA 103: 6315–6320 16606824 10.1073/pnas.0601755103 PMC1458875

[bib9] Feynman R (1959) http://www.zyvex.com/nanotech/feynman.html

[bib10] Fritz J, Baller MK, Lang HP, Rothuizen H, Vettiger P, Meyer E, Guntherodt H, Gerber C, Gimzewski JK (2000) Translating biomolecular recognition into nanomechanics. Science 288: 316–318 10764640 10.1126/science.288.5464.316

[bib11] Hansen KM, Ji HF, Wu G, Datar R, Cote R, Majumdar A, Thundat T (2001) Cantilever-based optical deflection assay for discrimination of DNA single-nucleotide mismatches. Anal Chem 73: 1567–1571 11321310 10.1021/ac0012748

[bib12] Harisinghani MG, Barentsz J, Hahn PF, Deserno WM, Tabatabaei S, van de Kaa CH, de la Rosette J, Weissleder R (2003) Noninvasive detection of clinically occult lymph-node metastases in prostate cancer. N Engl J Med 348: 2491–2499 12815134 10.1056/NEJMoa022749

[bib13] Hirsch LR, Stafford RJ, Bankson JA, Sershen SR, Rivera B, Price RE, Hazle JD, Halas NJ, West JL (2003) Nanoshell-mediated near-infrared thermal therapy of tumors under magnetic resonance guidance. Proc Natl Acad Sci USA 100: 13549–13554 14597719 10.1073/pnas.2232479100 PMC263851

[bib14] Hood JD, Bednarski M, Frausto R, Guccione S, Reisfeld RA, Xiang R, Cheresh DA (2002) Tumor regression by targeted gene delivery to the neovasculature. Science 296: 2404–2407 12089446 10.1126/science.1070200

[bib15] Horber JK, Miles MJ (2003) Scanning probe evolution in biology. Science 302: 1002–1005 14605360 10.1126/science.1067410

[bib16] Jiang W, Papa E, Fischer H, Mardyani S, Chan WC (2004) Semiconductor quantum dots as contrast agents for whole animal imaging. Trends Biotechnol 22: 607–609 15542145 10.1016/j.tibtech.2004.10.012

[bib17] Lidke DS, Nagy P, Heintzmann R, Arndt-Jovin DJ, Post JN, Grecco HE, Jares-Erijman EA, Jovin TM (2004) Quantum dot ligands provide new insights into erbB/HER receptor-mediated signal transduction. Nat Biotechnol 22: 198–203 14704683 10.1038/nbt929

[bib18] McDevitt MR, Ma D, Lai LT, Simon J, Borchardt P, Frank RK, Wu K, Pellegrini V, Curcio MJ, Miederer M, Bander NH, Scheinberg DA (2001) Tumor therapy with targeted atomic nanogenerators. Science 294: 1537–1540 11711678 10.1126/science.1064126

[bib19] Moghimi SM, Hunter AC, Murray JC (2005) Nanomedicine: current status and future prospects. FASEB J 19: 311–330 15746175 10.1096/fj.04-2747rev

[bib20] Piner RD, Zhu J, Xu F, Hong S, Mirkin CA (1999) Dip Pen Nanolithography. Science 283: 661–663 9924019 10.1126/science.283.5402.661

[bib21] Rosi NL, Mirkin CA (2005) Nanostructures in biodiagnostics. Chem Rev 105: 1547–1562 15826019 10.1021/cr030067f

[bib22] Satchi-Fainaro R, Puder M, Davies JW, Tran HT, Sampson DA, Greene AK, Corfas G, Folkman J (2004) Targeting angiogenesis with a conjugate of HPMA copolymer and TNP-470. Nat Med 10: 255–261 14981512 10.1038/nm1002

[bib23] Sengupta S, Eavarone D, Capila I, Zhao G, Watson N, Kiziltepe T, Sasisekharan R (2005) Temporal targeting of tumour cells and neovasculature with a nanoscale delivery system. Nature 436: 568–572 16049491 10.1038/nature03794

[bib24] Stroh M, Zimmer JP, Duda DG, Levchenko TS, Cohen KS, Brown EB, Scadden DT, Torchilin VP, Bawendi MG, Fukumura D, Jain RK (2005) Quantum dots spectrally distinguish multiple species within the tumor milieu *in vivo*. Nat Med 11: 678–682 15880117 10.1038/nm1247 PMC2686110

[bib25] Voura EB, Jaiswal JK, Mattoussi H, Simon SM (2004) Tracking metastatic tumor cell extravasation with quantum dot nanocrystals and fluorescence emission-scanning microscopy. Nat Med 10: 993–998 15334072 10.1038/nm1096

[bib26] Wu G, Datar RH, Hansen KM, Thundat T, Cote RJ, Majumdar A (2001) Bioassay of prostate-specific antigen (PSA) using microcantilevers. Nat Biotechnol 19: 856–860 11533645 10.1038/nbt0901-856

[bib27] Xu JP, Ji J, Chen WD, Shen JC (2005) Novel biomimetic polymersomes as polymer therapeutics for drug delivery. J Control Release 107: 502–512 16154659 10.1016/j.jconrel.2005.06.013

[bib28] Yuan F, Leunig M, Huang SK, Berk DA, Papahadjopoulos D, Jain RK (1994) Microvascular permeability and interstitial penetration of sterically stabilized (stealth) liposomes in a human tumor xenograft. Cancer Res 54: 3352–3356 8012948

[bib29] Zheng G, Patolsky F, Cui Y, Wang WU, Lieber CM (2005) Multiplexed electrical detection of cancer markers with nanowire sensor arrays. Nat Biotechnol 23: 1294–1301 16170313 10.1038/nbt1138

